# Consumer Wearable Devices for Activity Monitoring Among Individuals After a Stroke: A Prospective Comparison

**DOI:** 10.2196/cardio.8199

**Published:** 2018-01-04

**Authors:** Gabriela M Rozanski, Anthony Aqui, Shajicaa Sivakumaran, Avril Mansfield

**Affiliations:** ^1^ Mobility Team Toronto Rehabilitation Institute-University Health Network Toronto, ON Canada; ^2^ Department of Physical Therapy University of Toronto Toronto, ON Canada; ^3^ Rehabilitation Sciences Institute University of Toronto Toronto, ON Canada; ^4^ Evaluative Clinical Sciences Hurvitz Brain Sciences Research Program Sunnybrook Research Institute Toronto, ON Canada

**Keywords:** physical activity, heart rate, accelerometry, stroke rehabilitation, walking

## Abstract

**Background:**

Activity monitoring is necessary to investigate sedentary behavior after a stroke. Consumer wearable devices are an attractive alternative to research-grade technology, but measurement properties have not been established.

**Objective:**

The purpose of this study was to determine the accuracy of 2 wrist-worn fitness trackers: Fitbit Charge HR (FBT) and Garmin Vivosmart (GAR).

**Methods:**

Adults attending in- or outpatient therapy for stroke (n=37) wore FBT and GAR each on 2 separate days, in addition to an X6 accelerometer and Actigraph chest strap monitor. Step counts and heart rate data were extracted, and the agreement between devices was determined using Pearson or Spearman correlation and paired *t* or Wilcoxon signed rank tests (one- and two-sided). Subgroup analyses were conducted.

**Results:**

Step counts from FBT and GAR positively correlated with the X6 accelerometer (ρ=.78 and ρ=.65, *P*<.001, respectively) but were significantly lower (*P*<.01). For individuals using a rollator, there was no significant correlation between step counts from the X6 accelerometer and either FBT (ρ=.42, *P*=.12) or GAR (ρ=.30, *P*=.27). Heart rate from Actigraph, FBT, and GAR demonstrated responsiveness to changes in activity. Both FBT and GAR positively correlated with Actigraph for average heart rate (*r*=.53 and .75, *P*<.01, respectively) and time in target zone (ρ=.49 and .74, *P*<.01, respectively); these measures were not significantly different, but nonequivalence was found.

**Conclusions:**

FBT and GAR had moderate to strong correlation with best available reference measures of walking activity in individuals with subacute stroke. Accuracy appears to be lower among rollator users and varies according to heart rhythm. Consumer wearables may be a viable option for large-scale studies of physical activity.

## Introduction

### Activity After Stroke

Physical activity and exercise are recommended for stroke survivors because of the wide range of benefits that support recovery [1,2]. In addition to reducing disability, fitness interventions, particularly cardiorespiratory training, improve walking ability and aerobic capacity [3,4]. On the basis of strong evidence for a clear effect on cardiovascular health [5], physical activity is a key component of secondary prevention to lower the risk of recurrent stroke [6]. Unfortunately, there is a gap between clinical guidelines and actual behavior. Studies have consistently found that persons with stroke are very sedentary [7-11], even when compared to older adults with other chronic health conditions [12,13]. Despite sufficient capability, many individuals are not active enough to support physical fitness [14], and cardiorespiratory health may decline over time [15].

### Monitoring Activity

As an outcome measure for research trials, for example, testing the effectiveness of exercise training, self-report measures are frequently used to collect information on free-living physical activity but are prone to inaccuracy (eg, overestimation) from recall bias [16,17]. As several investigators have recommended [14,17-19], combining objective methods of quantifying movement (ie, accelerometry) with questionnaires and heart rate monitors will allow for better evaluation of interventions, both in and outside of clinical settings (eg, rehabilitation). Wearable technology provides a practical way to continuously record physiological responses as daily activity is tracked; however, challenges to implementation exist in certain contexts such as with patient populations.

Historically, accelerometer-based activity monitors developed for research settings have been expensive and relatively difficult to use. For example, the Accelerometry for Bilateral Lower Extremities system, which accurately measures walking activity after stroke, requires trained personnel and a custom algorithm that operates on proprietary software to process the data [20]. The commercially available Actigraph wGT3X+ can measure heart rate with a chest strap sensor; however, adherence to wearing the device in the community is extremely low [19]. Activity monitoring is now accessible to the public with recently developed “fitness trackers” (eg, Fitbit and Garmin) that are inexpensive and user-friendly. These popular consumer devices provide information on step counts and heart rate and are, therefore, attractive for large-scale studies of physical activity. As a health behavior change strategy, feedback to the wearer could also foster motivation and accountability in self-management programs [21,22].

Accelerometers can be reliable and valid for activity monitoring in persons with stroke [9], whereas the measurement properties of consumer “wearables” have not been established. The purpose of this study was to determine the accuracy of 2 fitness trackers—Fitbit Charge HR (FBT) and Garmin Vivosmart (GAR)—for measuring physical activity among individuals attending stroke rehabilitation. We hypothesized that step counts and heart rate data from the consumer devices would have “acceptable” agreement with previously validated sensors. Patient perceptions of device acceptability and usability were also investigated.

## Methods

### Participants

This study was approved by the institutional research ethics board. Sample size target was 40 to represent the range of physical function typical of the subacute stroke population. Between June 2016 and March 2017, 37 adults attending in- or outpatient therapy for stroke at the Toronto Rehabilitation Institute provided written informed consent following an invitation to participate. Individuals were excluded if they were unable to walk without physical assistance from another person or if they were unable to understand written or spoken English. Participant characteristics are presented in Table 1.

### Procedures

Participants wore 4 devices for 5.5-10 hours consecutively: (1) Actigraph chest strap heart rate monitor worn under clothing; (2) wGT3X+ sensor (Actigraph, Pensacola, Florida, USA); (3) Model X6-2mini (“X6”) accelerometer (Gulf Coast Data Concepts, LLC, Waveland, Mississippi, USA); and (4) consumer wearable device on the wrist of the less-affected arm: FBT (Fitbit Inc., San Francisco, California, USA) and GAR (Garmin Ltd., Schaffhausen, Switzerland), which were worn on 2 separate days within 1 week. The wGT3X+ sensor is also capable of accelerometry but was only used in this study to store the Actigraph heart rate data. Although body location differs between devices, the placements are consistent with previous validation methods and regular functionality such that results are applicable to use in “real-life.”

Study personnel visited inpatients on the stroke unit in the morning (typically between 8 am and 9 am) to don the devices and then retrieved them at the end of the workday (~4 pm). Outpatient participants were met during the day and sent home wearing the devices, along with instructions to remove them before bed and to return them at their next visit or therapy session. A piece of Fabrifoam was used to affix the wGT3X+ sensor and X6 accelerometer to the ankle of the less-affected leg. Participants were instructed to go about normal daily activities and not remove the devices unless required (eg, discomfort, personal hygiene, or risk of damage), or unless they became a burden. Upon retrieval or return, participants completed a feasibility questionnaire asking about their experience and thoughts on the device (Multimedia Appendix 1). Part A was completed after each time either the FBT or GAR was worn, and Part B, pertaining to the chest strap and ankle units, was administered on the second day. Both parts consisted of 6 questions, and responses were obtained through yes/no options, space for open explanations, as well as Likert and visual analog scales.

**Table 1:** Participant characteristics (n=37).

Descriptive variable		Mean (SD)^a^, median, or count	Range or percentage^b^
Age, years		64.4 (15.0)	41-90
Women		13	35
Height, cm		171.0 (9.3)	152-190.5
Weight, kg		77.1 (15.4)	45-113
Time post stroke, days		42.6 (33.2)	12-135
**Affected side**			
	Left	20	54
	Right	15	41
	Bilateral	1	3
	None	1	3
NIH-SS^c^ score		2	0-11
COVS^d^ score		85	65-91
BBS^e^ score		53	4-56
CMSA^f^ stage of leg		6	4-7
CMSA^f^ stage of foot		6	3-7
Walking speed, m/s		0.92 (0.29)	0.28-1.5
**Gait aid**			
	None	12	32
	Rollator	17	46
	Single point cane	8	22
Atrial fibrillation		6	16

^a^SD: standard deviation.

^b^Percentages may not sum to 100% due to rounding.

^c^NIH-SS: National Institutes of Health-Stroke Scale.

^d^COVS: Clinical Outcome Variables Scale.

^e^BBS: Berg Balance Scale.

^f^CMSA: Chedoke-McMaster Stroke Assessment.

Within 2 days of activity monitoring, the following tests were conducted for each participant during a short data collection session or as part of routine clinical care (data then extracted from patient chart): the National Institutes of Health-Stroke Scale (NIH-SS) [23], Berg Balance Scale (BBS) [24], Clinical Outcome Variables Scale (COVS) [25], Chedoke-McMaster Stroke Assessment (CMSA) [26] stage of leg and foot, and self-selected walking pace obtained from a pressure-sensitive mat (GAITRite, CIR Systems Inc., Havertown, Pennsylvania, USA). Height and weight were also recorded. The following information was obtained from hospital charts or directly from participants: age, sex, time since stroke, lesion location, medical history, and list of medications.

Maximum heart rate was determined in 1 of the following 3 ways: cardiopulmonary exercise test as part of routine care (value recorded by electrocardiography when respiratory exchange ratio >1.1; n=3); estimation using published formulas (164−0.7×age for individuals taking beta-blockers [27] and 208−0.7×age for all others [28]; n=3 and 26, respectively); or peak heart rate observed during the 2-day monitoring if higher than the age-predicted maximum (n=5).

### Data Processing

Step counts were extracted from the X6 accelerometer data using a previously validated custom written algorithm implemented in MATLAB (MathWorks, Nantick, Massachusetts, USA) [29]. FBT and GAR were synchronized to the manufacturers’ Web-based applications to extract step counts.

Heart rate data, transmitted from the Actigraph chest strap monitor to the wGT3X+ sensor via Bluetooth, were transferred to a computer, initially processed in 60-second epochs using the ActiLife software version 6 (Actigraph, Pensacola, Florida, USA), and exported to a text file. We noted that a number of Actigraph data points were physiologically improbable (<45 beats per minute); these were removed before further processing. To allow for comparison of heart rate measurement, we created time-aligned Actigraph and FBT/GAR data series. Actigraph heart rate data were averaged over 5-min epochs to compare with FBT values, which were manually transcribed into a spreadsheet from the Web application due to manufacturer’s restrictions in accessing raw data. GAR heart rate time series data were downloaded from the Web application as TCX files in 60-second epochs and converted into text files.

Because a large amount of heart rate data were missing, we first examined responsiveness of Actigraph, FBT, and GAR measures to changes in activity. Step counts from the X6 accelerometer were tallied over 5-min (for FBT) and 1-min (for GAR) epochs and aligned with the heart rate data. Heart rate, as recorded by each device, was averaged over all periods of rest (epochs with zero steps recorded) and at 3 intensities of walking activity: 50-79%, 80-99%, and ≥100% of comfortable cadence (based on self-selected walking on the GAITRite mat). We then determined agreement between the Actigraph and FBT/GAR heart rate data. If epochs were missing for one device, the corresponding data were deleted for the other device. From these modified time series, average heart rate and time within a target zone (55-80% of maximum heart rate) were calculated for each device.

### Data Analysis

Step counts from FBT and GAR were compared with the X6 accelerometer using Spearman correlation (ρ) and Wilcoxon signed rank tests. Both step count analyses were conducted for the whole group and separately by usual gait aid. To test the responsiveness of the devices to changes in physical activity, average heart rate at each intensity was compared with resting heart rate using paired *t* tests. Pearson or Spearman correlation and paired *t* tests or Wilcoxon signed rank tests, respectively, were used to compare average heart rate and time within the target heart rate zone from the Actigraph with those from the FBT and GAR. Analyses of heart rate data were conducted for the group as a whole and separately for those with and without diagnosis of arrhythmia (ie, atrial fibrillation); only those participants with at least 60 min of valid heart rate data for both devices were included. For one-sided tests of equivalence, ±5% of the mean or median reference value was chosen as the upper/lower boundary limit; however, a clinically acceptable level of uncertainty is not clear from previous research. All statistical analyses were conducted in SAS version 9.2 (SAS Institute Inc., Cary, North Carolina, USA), and alpha was .05.

Bland-Altman plots were used to visualize interdevice agreement. The difference between the FBT or GAR data and the reference measurements for each participant were plotted against the average of the 2 values. The mean or median difference and its 95% CI or interquartile range were represented by lines on each graph.

## Results

### Missing Data

Out of all the participants, 5 chose not to complete the second day of the study; therefore, analysis of step count data was limited to 36 participants for FBT and 33 for GAR. Furthermore, 2 participants declined to wear the Actigraph chest strap, and there were less than 60 min of valid heart rate data on both devices for 4 (when wearing FBT) and 9 participants (when wearing GAR); therefore, comparison of Actigraph and wrist-device heart rate data was limited to 30 (for FBT) and 22 (for GAR) participants. Potential reasons for missing data are discussed below.

### Step Counts

**Figure 1:**
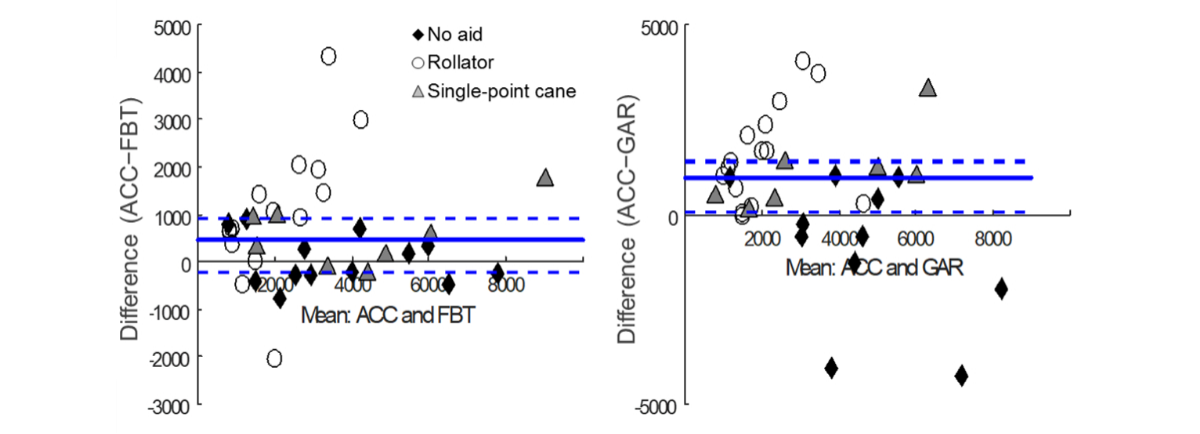
Bland-Altman plots of step count agreement between X6 accelerometer (ACC) and wrist-worn devices: left, Fitbit Charge HR (FBT), and right, Garmin Vivosmart (GAR). Solid bold line is the median difference between step count measurements, averaged over all participants. Dashed lines are the interquartile range of the difference. Note that the scale on the y-axis is not the same between the graphs.

Results of the step count analyses are presented in Table 2, and Bland-Altman plots showing agreement of each wrist-worn device with the X6 accelerometer are in Figure 1.

**Table 2:** Agreement in step counts between X6 accelerometer and wrist-worn devices.

Group	FBT^a^					GAR^b^				
	n	ρ (*P* value)	Difference^c^ (IQR)^d^	Error (IQR)	*P*; *P*_U/L_^e^	n	ρ (*P* value)	Difference (IQR)	Error (IQR)	*P*; *P*_U/L_
All participants	36	.78 (<.001)	463 (−220 to 924)	32.9% (8.3-52.5)	.002; .99	33	.65 (<.001)	963 (59-1390)	40.9% (14.9-69.5)	.008; break/>.99
No gait aid	13	.97 (<.001)	−203 (−288 to 339)	10.3% (5.5-30.4)	.85; .32	11	.56 (.07)	−561 (−1960 to 971)	23.1% (14.7-46.1)	.28; break/>0.77
Rollator	15	.42 (.12)	926 (386-1939)	52.3% (39.9-56.9)	.01; .99	15	.30 (.27)	1390 (283-2376)	67.2% (26.2-73.0)	.001; break/>>.99
Single-point cane	8	.98 (<.001)	406 (−18 to 912)	12.6% (5.8-22.3)	.08; .90	7	.93 (.003)	963 (377-1330)	21.3% (15.0-41.1)	.02; break/>.99

^a^FBT: Fitbit Charge HR.

^b^GAR: Garmin Vivosmart.

^c^The difference is calculated as the X6 accelerometer step count minus the wrist device step count; therefore, a positive value means the wrist-worn device undercounted, whereas a negative value means the wrist-worn device overcounted.

^d^IQR: interquartile range

^d^*P* values are for Wilcoxon signed rank tests: two-sided to compare median step counts of the devices and one-sided testing of whether the median difference is significantly different from a lower (*P*_L_) and upper (*P*_U_) limit (the greater of the 2 is reported).

For step count measurement of all participants combined, there was a strong positive correlation between the X6 accelerometer and FBT (ρ=.78, *P*<.001) and a moderate positive correlation between the X6 accelerometer and GAR (ρ=.65, *P*<.001). However, the FBT (S_35_=191.5, *P*=.002; 32.9% error) and GAR (S_32_=144.5, *P*=.008; 40.9% error) significantly undercounted steps compared with the X6 accelerometer. According to the equivalence tests, the median step count differences were not significantly less than the respective upper boundary limits (*P*_U_=.99).

For the gait aid subanalyses, there were strong positive correlations between X6 accelerometer and FBT step counts (ρ>.97, *P* values <.001) and no significant difference in step counts between devices in both the no gait aid (S_12_=−3, *P*=.85; 10.3% error) and single-point cane (S_7_=13, *P*=.08; 12.6% error) groups. However, nonequivalence from undercounting was revealed by one-tailed tests (*P*_U_=.32 and *P*_U_=.90, respectively). Conversely, for the rollator group, there was no significant correlation between X6 accelerometer and FBT step counts (ρ=.42, *P*=.12), and the FBT significantly undercounted steps compared with the X6 accelerometer (S_14_=44, *P*=.01; *P*_U_=.99; 52.3% error). There was a moderate positive correlation (ρ=.56, *P*=.07) and no significant difference in step counts between the X6 accelerometer and GAR (S_10_=−13, *P*=.28; 23.1% error) for participants not using a gait aid, but measurements were not equivalent (*P*_L_=.77). Although there was a strong positive correlation between step counts from the X6 accelerometer and GAR in the single-point cane group (ρ=.93, *P*=.003), the GAR significantly undercounted steps for these individuals (S_6_=14, *P*=.02; *P*_U_=.99; 21.3% error). In the rollator group, there was no significant correlation between X6 accelerometer and GAR step counts (ρ=.30, *P*=.27), and the GAR significantly undercounted steps compared with the X6 accelerometer (S_14_=59, *P*=.001; *P*_U_>.99; 67.2% error).

### Heart Rate

On average, valid Actigraph, FBT, and GAR heart rate data were available for 42.4% (95% CI 35.7-48.8), 95.3% (95% CI 93.3-97.2), and 75.1% (95% CI 63.8-86.5) of the time worn during monitoring, respectively. Data indicating responsiveness of heart rate to changes in activity are presented in Table 3.

**Table 3:** Responsiveness of heart rate devices to change in walking activity.

Cadence (%)	Five-min epochs				One-min epochs			
	FBT^a^		Actigraph		GAR^b^		Actigraph	
	Percentage change (95% CI)^c^	*P* (n)^d^	Percentage change (95% CI)	*P* (n)	Percentage change (95% CI)	*P* (n)	Percentage change (95% CI)	*P* (n)
50-79	6.3 (3.2-9.3)	<.001 (27)	4.7 (0.6-8.8)	.03 (20)	2.8 (0.9-4.7)	.006 (30)	3.5 (0.7-6.3)	.02 (28)
80-99	15.4 (10.0-20.7)	<.001 (13)	11.8 (−0.2 to 23.7)	.05 (7)	3.5 (0.5-6.5)	.02 (26)	6.6 (3.8-9.5)	<.001 (25)
≥100	16.8 (7.9-25.8)	.005 (6)	17.8 (−26.3 to 62.0)	.22 (3)	1.8 (−1.6 to 5.3)	.27 (14)	12.9 (5.7-20.2)	.003 (9)

^a^FBT: Fitbit Charge HR.

^b^GAR: Garmin Vivosmart.

^c^Values presented are mean increase in heart rate from rest (“percentage change”), expressed as a percentage of estimated maximum heart rate, with 95% CI in parentheses.

^d^*P* values correspond to *t* tests comparing heart rate at rest and different cadences. Note the sample sizes (n) differ between comparisons as not all participants walked at each cadence, or there were no valid heart rate data at that activity level.

Both Actigraph (for 1-min epochs) and FBT showed significant increases in heart rate when participants walked at greater than or equal to 50% of their self-paced cadence compared with rest (*P* values≤.017). When Actigraph heart rate data were averaged over 5-min epochs, there was a trend toward higher heart rate with increasing activity, but the lack of significance for walking at 80-99% and greater than or equal to 100% of self-paced cadence was likely due to a large amount of missing data and consequent low sample size (n=7 and n=3, respectively; *P* values≥.052). GAR showed a significant increase in heart rate at 50-79% (*P*=.006) and 80-99% (*P*=.02) but not greater than or equal to 100% of self-paced cadence (*P*=.27), compared with rest.

**Figure 2:**
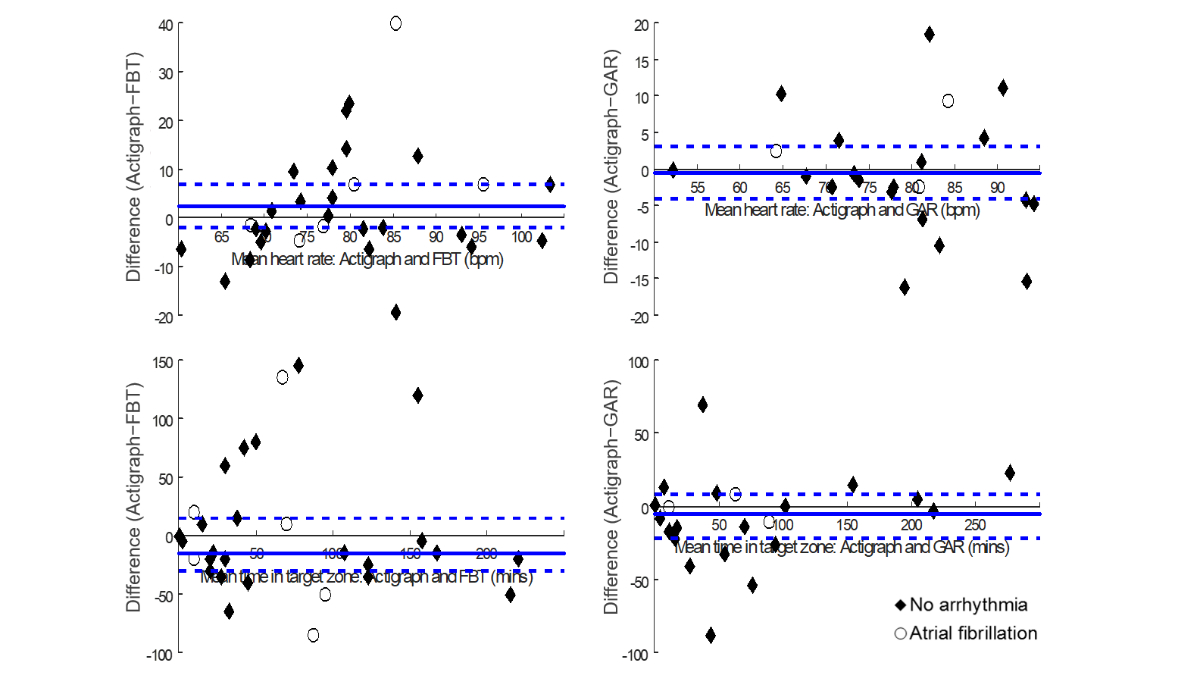
Bland-Altman plots of agreement between Actigraph and wrist-worn devices: left, Fitbit Charge HR (FBT), and right, Garmin Vivosmart (GAR) for mean heart rate (top) and median time in target zone (bottom). Solid bold line is the mean difference between measurements, averaged over all participants. Dashed lines are the 95% CI or interquartile range of the difference. Note that the scale on the y-axis is not the same between the graphs.

Results of the comparison in heart rate data between devices are presented in Table 4, and Bland-Altman plots showing agreement between the wrist-worn devices and Actigraph are in Figure 2. For average heart rate of all participants, there were moderate positive correlations and no significant difference between Actigraph and FBT (*r*=.53, *P*=.003; *t*_29_=1.11, *P*=.28; 10.1% error) as well as between GAR and Actigraph (*r*=.75, *P*<.001; *t*_21_=−0.28, *P*=.78; 7.4% error); however, nonequivalence was revealed by one-tailed tests (*P*_U_=.30 and *P*_L_=.16, respectively). There was a significant positive correlation of Actigraph with GAR (ρ=.74, *P*<.001) and FBT (ρ=.49, *P*=.006) for time in target zone. Time in target zone was not significantly different between FBT and Actigraph (S_29_=−37.5, *P*=.43; 42.9% error) or between GAR and Actigraph (S_21_=−42, *P*=.15; 28.4% error), but measurements were not equivalent (*P*_L_=.67 and *P*_L_=.82, respectively).

When participants without arrhythmia were analyzed separately, the correlation between Actigraph and FBT for average heart rate (*r*=.64, *P*<.001) and time in target zone (ρ=.57, *P*=.004) improved slightly. Conversely, there were no significant correlations between Actigraph and FBT for average heart rate (*r*=.16, *P*=.77) or time in target zone (ρ=−.03, *P*=.96) among participants with atrial fibrillation. Average heart rate and time in target zone were not significantly different between FBT and Actigraph for participants without arrhythmia or those with atrial fibrillation (*P* values>.31; 9.9-66.7% error). According to the equivalence tests, none of the interdevice differences were significantly less than the upper or lower boundary limits (range of *P* values=.20-.70). 

**Table 4:** Agreement in heart rate data between Actigraph and wrist-worn devices.

Group		FBT^a^					GAR^b^				
		n	Correlation coefficient (*P* value)	Difference^c^ (95% CI) or (IQR)^d^	Error (95% CI) or (IQR)	*P*; *P*_U/L_^e^	n	Correlation coefficient (*P* value)	Difference (95% CI) or (IQR)	Error (95% CI) or (IQR)	*P*; *P*_U/L_
**Average heart rate, beats per minute** ^f^											
	All participants	30	.53 (.003)	2.4 (−2.0 to 6.8)	10.1% (6.9-13.3)	.28; .30	22	.75 (<.001)	−0.5 (−4.1 to 3.1)	7.4% (4.7-10.2)	.78; .16
	No arrhythmia	24	.64 (<.001)	1.1 (−3.2 to 5.5)	9.9% (6.8-13.1)	.61; .20	19	.74 (<.001)	−1.1 (−5.2 to 3.0)	7.7% (4.5-10.8)	.59; .23
	Atrial fibrillation	6	.16 (.77)	7.6 (−9.8 to 24.9)	10.7% (−0.1 to 21.5)	.31; .67	3	.87 (.33)	3.1 (−11.6 to 17.8)	5.8% (1.2-10.4)	.46; .47
**Time in target zone, minutes** ^g^											
	All participants	30	.49 (.006)	−15 (−30, 15)	42.9% (28.6-109.5)	.43; .67	22	.74 (<.001)	−5.5 (−22 to 8)	28.4% (15.7-49.5)	.15; .82
	No arrhythmia	24	.57 (.004)	−15 (−28 to 12)	42.9% (28.6-100.0)	.45; .70	19	.73 (<.001)	−8 (−26 to 9)	29.4% (16.7-57.8)	.16; .85
	Atrial fibrillation	6	−.03 (.96)	−5 (−50 to 20)	66.7% (38.1-145.2)	.88; .58	3	1.0 (<.001)	−1 (−11 to 8)	15.7% (8.8-18.6)	.75; .38

^a^FBT: Fitbit Charge HR.

^b^GAR: Garmin Vivosmart.

^c^The difference is calculated as the Actigraph value minus the value for the wrist device; therefore, a positive value means the wrist-worn device underestimated, whereas a negative value means the wrist-worn device overestimated.

^d^IQR: interquartile range.

^e^*P* values are for paired *t* or Wilcoxon signed rank tests: two-sided to compare means or medians of the devices and one-sided testing of whether the mean or median difference is significantly different from a lower (*P*_L_) and upper (*P*_U_) limit (the greater of the 2 is reported).

^f^Pearson correlation coefficient.

^g^Spearman correlation coefficient.

The positive correlations between Actigraph and GAR for average heart rate and time in zone remained high for both those without arrhythmia (*r*=.74, *P*<.001 and ρ=.73, *P*<.001, respectively) and those with atrial fibrillation (*r*=.87, *P*=.33 and ρ=1.0, *P*<.001, respectively). Average heart rate and time in target zone were not significantly different between GAR and Actigraph for participants without arrhythmia or those with atrial fibrillation (*P* values≥.16; 5.8-29.4% error), yet there was no equivalence of measurements (range of *P* values=.16-.85).

### Device Acceptability and Usability

All participants completed the feasibility questionnaire for at least 1 device; 27 individuals evaluated their experience with all 4 devices (both fitness trackers, X6 accelerometer, and chest strap). In terms of comfort, 94% (31/33) and 97% (32/33) of participants found FBT and GAR, respectively, to be somewhat or very comfortable, whereas 89% (33/37) and 91% (31/34) said the same for the X6 accelerometer and heart rate monitor, respectively. Overall, 7 individuals reported problems wearing the devices. Issues included general discomfort, trouble with doffing, and wrist strap feeling too tight. When asked about the level of confidence in their ability to don and doff independently, the average response, based on a visual analog scale from 0 (not confident at all) to 10 (extremely confident), was 8.8 for all devices except for the chest strap (7.7). A large majority of participants said they would be likely or very likely to participate in a study that involved wearing the FBT (28/33, 85%), GAR (29/33, 88%), X6 accelerometer (30/37, 81%), or heart rate monitor (24/34, 71%) every day for 1 week. Some concerns included sleeping with the device and remembering to put it on.

## Discussion

### Principal Findings

The main finding of this study is that FBT and GAR had a moderate to strong correlation with the best available reference devices for measuring walking activity in terms of step count and heart rate among individuals with subacute stroke. Accuracy varied widely according to mobility status and based on whether or not heart rhythm was normal. The consumer devices were well accepted by participants.

### Counting Steps

In patients not using a gait aid, steps counted by the fitness trackers were not different from that of the X6 accelerometer; however, GAR was inaccurate (23.1% error) compared with FBT (10.3% error), and equivalence was not demonstrated. Neither device appears suitable for rollator users due to significant undercounting, and despite strong correlation of FBT with the X6 accelerometer for single-point cane users, accuracy was low (>10% error). Most studies on consumer wearables have been conducted with healthy adults, but, consistent with our results, overall validity of step counts with a tendency toward underestimation has been found [30]. A different Fitbit model worn at the waist also undercounted steps in people with chronic stroke tested over a short distance in a closed environment [31]. The lower accuracy in gait aid users seen herein may be attributed to a slower walking speed, which has been shown to affect accelerometer step count, especially when placed at the hip [32-34]. Limited arm swing during ambulation with a rollator could also limit accuracy of wrist-worn devices. Alternative placements of consumer devices for specific clinical subgroups should be explored in future studies.

### Measuring Heart Rate

Although the high accuracy of chest strap monitors is well established, comparison of heart rate data was complicated by the low reliability of the Actigraph acquisition system. This may have been due to Bluetooth transmission problems, drying of electrode areas over time, or chest strap placement issues such as slippage through the day. Therefore, the positive correlations of the Actigraph with FBT and GAR could be over- or underestimated, and power to detect a difference in average heart rate or time in target zone was reduced. Considering data availability and responsiveness, FBT appeared superior, but heart rate measured by GAR was also sensitive to changes in walking intensity (ie, cadence). In general, intensity of physical activity appeared to be relatively low according to total time in target zone and the sample size of higher cadence levels, although data may not have been available when participants walked quickly, which could account for the relatively high error associated with this parameter. For individuals with atrial fibrillation, FBT had lower agreement with the Actigraph (10.7% average heart rate error) than did GAR; however, it is not clear to which device the inaccuracy for this clinical subgroup can be attributed as no criterion standard was performed for comparison (see below). Some wearable heart rate monitors based on photopletysmography (optical detection of blood volume changes) have been evaluated with evidence of variable accuracy [35-38]. In 2 studies with healthy adults, the FBT was found to correlate well with electrocardiography but underestimated heart rate during more vigorous physical exertion [35,37]. The same device was significantly less accurate among hospital inpatients who were not in sinus rhythm [36]. Therefore, this technology may be more reliably applied in a clinical context when exercise intensity is limited and no arrhythmias are present.

### Strengths and Limitations

It may have been possible to minimize the large amount of missing data by performing the study in a laboratory with constant supervision of the participants, but, aside from being more resource-efficient, our design benefits from ecological validity. Devices were compared from different but typical and realistic body positions. The portability of the technology tested allowed for monitoring to take place under free-living conditions over many hours such that a range of activity levels could theoretically be captured. This precluded the use of “gold standard” measures such as electrocardiography and visual step counts to establish criterion validity. Clinically relevant variables were evaluated, and subanalyses revealed differences between groups to more precisely guide the interpretation of results. Although manual entry of some data was necessary for the purpose of this study, the commercially intended functions of consumer wearables provide information in a user-friendly format that could easily be applied in clinical or research settings.

### Conclusions

Overall, the strength of correlations and measures of accuracy suggest that FBT is valid for step-counting in individuals who do not use a gait aid, whereas both devices are suitable for group analyses that tolerate greater measurement variability. The tendency to underestimate steps and general lack of equivalence with reference standards should be considered. FBT was reliable, responsive, and accurate for recording nonarrhythmic heart rate. Assessing validity in participants with atrial fibrillation was limited by low sample sizes. These results, along with the generally positive feedback from the feasibility questionnaire, imply that fitness trackers may be a viable alternative to “research grade” activity monitors for large clinical trials. As more commercial models and algorithms are developed, consumer wearables should continue to be investigated for accuracy. The selection of a device for research or health care purposes will ultimately depend on the context, including patient population and primary outcome.

## Appendix

Multimedia Appendix 1 Feasibility questionnaire.

## Abbreviations

BBS: Berg Balance Scale
CMSA: Chedoke-McMaster Stroke Assessment
COVS: Clinical Outcome Variables Scale
FBT: Fitbit Charge HR
GAR: Garmin Vivosmart
NIH-SS: National Institutes of Health-Stroke Scale
SD: standard deviation
