# Supplementary material for: Consumer Wearable Devices for Activity Monitoring Among Individuals After a Stroke: A Prospective Comparison
Source: JMIR Cardio. 2018 Jan 4;2(1):e1. doi: 10.2196/cardio.8199 (PMC6834221; doi:10.2196/cardio.8199)
Supplement: Multimedia Appendix 1 [file cardio_v2i1e1_app1.pdf]

Date:_____________________________

Investigator initials: ___________

Feasibility questionnaire

Instructions for administrator: show devices to participant throughout the questionnaire to remind them what each device is when you ask about it.

Part A – consumer device

Consumer device worn on this day:
- ❑ FitBit Charge HR
- ❑ Garmin Vivosmart

1. How comfortable was it for you to wear the **wrist-worn device** during 1 day? *Please choose the best answer.*

Not very comfortable	Not comfortable	Neutral	Somewhat comfortable	Very comfortable
❑	❑	❑	❑	❑

2. Did you have any problems wearing the devices?
   - ❑ No
   - ❑ Yes

   If Yes, please explain your answer: ____
   ____
   ____
   ____
   ____

3. These kinds of devices are usually used to measure physical activity over several days. How likely would you be to participate in a study where you had to wear the device every day for 1 week?

Very unlikely	Unlikely	Maybe	Likely	Very likely
❑	❑	❑	❑	❑

4. Do you think you would have any problems or discomforts with wearing the device every day for a week?
   Please choose the best answer.

Very unlikely	Unlikely	Maybe	Likely	Very likely
❑	❑	❑	❑	❑

If you think there might be any problems or discomforts with wearing the devices on the daily basis, please explain your answer:____
____
____

5. Do you think you would have any problems or discomforts sleeping while wearing the device? *Please choose the best answer.*

Very unlikely	Unlikely	Maybe	Likely	Very likely
❑	❑	❑	❑	❑

If you think there might be any problems or discomforts with sleeping while wearing the device, please explain your answer:____
____
____
____

6. How confident are you that you would be able to put on and take off the **wrist-worn device** on your own if you were given the device to wear at home?
0= not confident at all; 10 = extremely confident

0 ———————————————— 10

Part B
Complete on last day only.

7. How comfortable was it for you to wear the **chest strap** and **accelerometer** during 1 day? *Please choose the best answer.*

	Not very comfortable	Not comfortabl e	Neutral	Somewhat comfortabl e	Very comfortabl e
Chest strap for 1 day	❑	❑	❑	❑	❑
Accelerometer worn at the ankle for 1 day	❑	❑	❑	❑	❑

8. Did you have any problems wearing the devices?
   - ❑ No
   - ❑ Yes

   If Yes, please explain your answer: ____
   ____
   ____
   ____
   ____
   ____

Date:_____________________________

Investigator initials: ___________

9. These kinds of devices are usually used to measure physical activity over several days. How likely would you be to participate in a study where you had to wear the:

	Very unlikely	Unlikely	Maybe	Likely	Very likely
Chest strap every day for 1 week?	❑	❑	❑	❑	❑
Accelerometer at the ankle every day for 1 week?	❑	❑	❑	❑	❑

10. Do you think you would have any problems or discomforts with wearing devices every day for a week?
Please choose the best answer.

	Very unlikely	Unlikely	Maybe	Likely	Very likely
Chest strap every day for 1 week?	❑	❑	❑	❑	❑
Accelerometer at the ankle every day for 1 week?	❑	❑	❑	❑	❑

If you think there might be any problems or discomforts with wearing the devices on the daily basis, please explain your answer:_______________________________________
____
____
____

11. How confident are you that you would be able to put on and take off the **chest strap** on your own if you were given the device to wear at home?
0= not confident at all; 10 = extremely confident

0 ____________________ 1 0 ____________________

12. How confident are you that you would be able to put on and take off the **accelerometer** on your own if you were given the device to wear at home?
0= not confident at all; 10 = extremely confident

0 ____________________ 1 0 ____________________
